# Afferent Connectivity of the Zebrafish Habenulae

**DOI:** 10.3389/fncir.2016.00030

**Published:** 2016-04-26

**Authors:** Katherine J. Turner, Thomas A. Hawkins, Julián Yáñez, Ramón Anadón, Stephen W. Wilson, Mónica Folgueira

**Affiliations:** ^1^Department of Cell and Developmental Biology, University College London (UCL)London, UK; ^2^Neurover Group, Centro de Investigacións Científicas Avanzadas (CICA) and Department of Cell and Molecular Biology, University of A Coruña (UDC)A Coruña, Spain; ^3^Department of Cell Biology and Ecology, Faculty of Biology, University of Santiago de CompostelaSantiago de Compostela, Spain

**Keywords:** habenula, connections, afferents, entopeduncular nucleus, posterior tuberculum, basal ganglia, zebrafish

## Abstract

The habenulae are bilateral nuclei located in the dorsal diencephalon that are conserved across vertebrates. Here we describe the main afferents to the habenulae in larval and adult zebrafish. We observe afferents from the subpallium, nucleus rostrolateralis, posterior tuberculum, posterior hypothalamic lobe, median raphe; we also see asymmetric afferents from olfactory bulb to the right habenula, and from the parapineal to the left habenula. In addition, we find afferents from a ventrolateral telencephalic nucleus that neurochemical and hodological data identify as the ventral entopeduncular nucleus (vENT), confirming and extending observations of Amo et al. (2014). Fate map and marker studies suggest that vENT originates from the diencephalic prethalamic eminence and extends into the lateral telencephalon from 48 to 120 hour post-fertilization (hpf). No afferents to the habenula were observed from the dorsal entopeduncular nucleus (dENT). Consequently, we confirm that the vENT (and not the dENT) should be considered as the entopeduncular nucleus “proper” in zebrafish. Furthermore, comparison with data in other vertebrates suggests that the vENT is a conserved basal ganglia nucleus, being homologous to the entopeduncular nucleus of mammals (internal segment of the globus pallidus of primates) by both embryonic origin and projections, as previously suggested by Amo et al. (2014).

## Introduction

The habenulae are components of a highly conserved circuit interconnecting areas of the forebrain with the midbrain and hindbrain (Sutherland, 1982; Bianco et al., 2008). The habenulae are implicated in a range of behaviors, including the modulation of fear and aversion, reproductive and maternal behaviors and sleep initiation and duration (Hong and Hikosaka, 2008; Hikosaka, 2010; Shabel et al., 2012). The habenulae also regulate monoaminergic activity and consequently have been implicated in schizophrenia, addiction and mood disorders (Hikosaka, 2010; Aizawa et al., 2011; Lecca et al., 2014). Mammalian habenulae are subdivided into medial and lateral nuclei that show different connection patterns (Herkenham and Nauta, 1977). The medial habenula receives major afferents from the septum, among other regions, and projects mainly to the interpeduncular nucleus (IPN; Herkenham and Nauta, 1977). The lateral habenula receives major afferents from the telencephalic entopeduncular nucleus (ENT) and projects mainly to the ventral tegmental area and median raphe, among other areas of the midbrain and hindbrain (Herkenham and Nauta, 1977; Hikosaka, 2010).

In zebrafish, descriptions of afferents to the habenular subnuclei and comparisons with other vertebrates are scanty (Amo et al., 2014). Zebrafish habenulae can be subdivided into dorsal and ventral nuclei, homologous to the mammalian medial and lateral habenula respectively (Amo et al., 2010). Thus far, connectional studies performed in zebrafish have mostly focused on habenula efferents to the midbrain and hindbrain (Aizawa et al., 2005; Bianco et al., 2008; Miyasaka et al., 2009; Amo et al., 2010), giving less attention to habenula afferents. Hendricks and Jesuthasan (2007) described afferents to the habenula from three nuclei in the zebrafish larva (pallium, eminentia thalami and posterior tuberculum), but correspondence with adult brain areas was not described. Tracing results in adults reported a projection from the ventral entopeduncular nucleus (vENT) to the ventral habenula (Amo et al., 2014). Other than that, only asymmetric projections from the olfactory bulb and parapineal have been reported (Concha et al., 2003; Miyasaka et al., 2009, 2014). Despite these studies, and by comparison with other teleosts (Villani et al., 1996; Yáñez and Anadón, 1996), our knowledge of habenular connectivity in the zebrafish is likely to be far from complete.

One striking feature of the zebrafish habenulae is that they exhibit left-right asymmetries, which are most prominent in the medial/dorsal habenula (Concha and Wilson, 2001; Roussigne et al., 2012; Hong et al., 2013; deCarvalho et al., 2014; Duboc et al., 2015). Although there is considerable variation in the extent of habenular asymmetry between vertebrate classes and species, it seems likely that asymmetries are present in all vertebrates (Concha and Wilson, 2001; Villalón et al., 2012) including mammals (e.g., Ichijo et al., 2015). Habenular asymmetry is reflected in both the efferent connectivity of the left and right habenulae (Aizawa et al., 2005; Gamse et al., 2005; Bianco et al., 2008; Amo et al., 2010) and in afferent input (Concha et al., 2003; Hendricks and Jesuthasan, 2007; Miyasaka et al., 2009, 2014). For instance, in zebrafish, parapineal axons only innervate the left habenula (Concha et al., 2003; Bianco et al., 2008; Regan et al., 2009) whereas a subset of olfactory bulb mitral cells only innervate the right habenula (Miyasaka et al., 2009, 2014).

This study describes the main afferents to the habenula in adult and larval zebrafish by using carbocyanine tract tracing experiments in combination with immunohistochemistry and analyses of transgenic lines in which neurons that project to the habenulae are labeled. In addition, we examine the development of what we find to be the main telencephalic nucleus projecting to the habenula: the vENT, which we propose is the homolog of the mammalian ENT as also suggested by Amo et al. (2014).

## Materials and Methods

### Fish Stocks and Maintenance

Adult zebrafish (*Danio rerio*, Cyprinidae) were maintained at University College London (UCL) Fish Facility under standard conditions: 28°C and 14 h light/10 h dark periods (Westerfield, 2000). Embryos were raised in fish water at 28°C and staged according to Kimmel et al. (1995). To avoid pigment formation in larvae, 0.003% w/v Phenylthiocarbamide (PTU, Sigma) was added to the fish water at 24 h post fertilization (hpf). All experimental procedures were conducted under licence from the UK Home Office, following UK Home Office regulations and/or European Community Guidelines on Animal Care and Experimentation and were approved by animal care and use committees.

The following zebrafish strains were used in this study: AB and TL wild types, *Tg(lhx5:GFP)*^b1205^ and *Tg(lhx5:Kaede)*^b1204^ (Gao et al., 2012; Zhang et al., 2012), *Tg(1.4dlx5a-dlx6a:GFP)*^ot1^ (Zerucha et al., 2000), *Tg(isl1:GFP)*^rw0^ (Higashijima et al., 2000), *Et(gata2:GFP)*^bi105^ (Folgueira et al., 2012), *Tg(slc17a6b:DsRed)*^nns9Tg^ (Miyasaka et al., 2009; Kani et al., 2010) and *Tg(−2.7shh:GFP)*^t10^ (Albert et al., 2003).

### Anesthesia and Fixation

Specimens were deeply anesthetized in 0.2% tricaine (MS222, Sigma) in fresh water and fixed in 4% paraformaldehyde (PFA) in phosphate buffered saline (PBS) with 4% sucrose either by immersion (embryos and larvae) or by transcardiac perfusion (adults). The tissue was then post-fixed in the same fixative for 24 h at room temperature. After this, cranial skin, jaw and other tissues were removed from embryos and larvae by fine dissection using two pairs of sharp forceps on embryos pinned in sylgard (Turner et al., 2014). After washing in PBS and dehydration in methanol, they were kept at −20°C for at least 24 h. Adult fish brains were dissected and kept either in PFA (for neural tracer application) or in PBS (for immunostaining and other purposes) at 4°C until use.

### Neural Tract Tracing

For each experiment, a tiny crystal of the lipophilic tracer DiI (Invitrogen) was placed on the tip of a sharp tungsten needle and inserted into the adult brain under a stereomicroscope. DiI applications to the right and left habenula (7 cases for right habenula/8 cases for left habenula) were performed approaching the brain from a dorsal aspect after removing the pineal and parapineal. Other brain structures accessed by this procedure were: olfactory bulb (2 cases), posterior area of the pallium (Dp; 2 cases), the vENT (2 cases) and the posterior hypothalamic lobe (1 case). The application area was sealed with melted 3% agarose and brains were incubated in the dark for 2–14 days in PFA at 37°C. After this period, transverse sections (50 μm thick) were cut on a vibratome, mounted on gelatin-coated slides and photographed using an epifluorescence photomicroscope (Nikon Eclipse 90i) equipped with an Olympus DP70 digital camera. Some selected sections were further imaged using a laser scanning confocal microscope (Nikon A1R).

### Nissl Staining

Serial transverse sections of the brain were stained with cresyl violet and used for studying cell architecture and distribution of different nuclei.

### Antibody Labeling

Embryos and larvae, dissected or otherwise, were stained as whole mounts following standard procedures (Shanmugalingam et al., 2000; Turner et al., 2014). In brief, specimens were rehydrated to phosphate buffered saline with 0.5% Triton X-100 (PBS-T) and after a brief proteinase digestion to improve permeability of the tissue, specimens were incubated with primary antibody overnight at 4°C. Specimens were then washed in PBS-T and incubated with fluorescent secondary antibodies again overnight at 4°C. After washing in PBS-T, embryos and larvae were mounted in 2% low-melting point agarose and imaged on a Leica confocal laser scanning system. For a detailed protocol on larval dissection, antibody labeling and mounting for imaging see Turner et al. (2014).

Adult brains were cryoprotected, frozen with liquid nitrogen-cooled methylbutane and cut in sections (12 μm thick) on a cryostat. After mounting the sections on gelatinized slides, they were rinsed in PBS-T and preincubated with normal goat serum (Sigma, 1:10) for 1 h. Next, they were incubated with a primary antibody or a cocktail of primary antibodies overnight at room temperature and then with secondary fluorescent antibodies for 1 h, followed by PBS washes. Sections were then mounted using glycerol-based mounting medium and photographed either under a laser scanning confocal microscope (Nikon A1R) or under a conventional epifluorescence microscope. For immunostaining against GFP after DiI application to the habenula, we followed the same procedure but avoided the use of detergents, as they would have washed away the lipophilic tracer.

One *Tg(lhx5:GFP)*^b1205^ adult brain was stained as whole mount. For this, we followed the same protocol as for larval whole mount immunostaining, but with longer proteinase digestion and primary antibody incubation (48 h).

Antibodies and dilutions used were as follows: neuropeptide Y (NPY, Sigma, Cat# N9528, dilution 1:1000), rabbit anti-green fluorescent protein (GFP, Torrey Pines Biolabs, Cat# TP401, dilution 1:1000), rat anti-GFP (Nacalai Tesque, Cat# GF090R, dilution 1:1000), mouse anti-acetylated tubulin antibody (IgG2b; α-tubulin, Sigma, Cat# T7451, dilution 1:250), rabbit anti-calretinin antibody (CR, Swant, Cat# 7697, dilution 1:1000), rabbit anti-γ-aminobutyric acid (GABA, Sigma, Cat# A2052, dilution 1:1000), mouse anti-synaptic vesicle protein 2 (IgG1; SV2, DSHB, Cat# AB 2315387, dilution 1:250), rabbit anti-DSRed antibody (Living Colours, Clontech, Cat# 632496, dilution 1:300), Alexa Fluor 488 (Invitrogen, Goat anti-Rabbit Cat# A-11034, Goat anti-Rat Cat# A-11006, Goat anti-Mouse Cat# A-11029, dilution all 1:200), Alexa Fluor 568 (Invitrogen, Goat anti-Mouse IgG Cat# A-11031, Goat anti-Mouse IgG2b Cat# A-21144, dilution all 1:200) and Alexa Fluor 633 (Invitrogen, Goat anti-Mouse IgG Cat# A-21052; Goat anti-Mouse IgG1 Cat# A-21126, dilution all 1:200). To detect anti-acetylated tubulin and anti-SV2 in the same sample, isotype-specific secondary antibodies were used: Alexa Fluor 568 (IgG2b) and Alexa Fluor 633 (IgG1).

### Wholemount Fluorescent *in situ* Hybridization (FISH)

The FISH protocol was adapted from Jülich et al. (2005). Digoxigenin probes were made by standard protocols and were detected using the anti-DIG POD antibody (Roche, 1:1000) and stained using Cy3-tyramide substrate (Perkin Elmer, 1:50 in amplification buffer). After staining, antibody labeling for GFP was performed as above using rabbit anti-green fluorescent protein (GFP, Torrey Pines Biolabs, 1:1000) and Goat anti-Rabbit Alexa Fluor 488 (Invitrogen, 1:200) without further PK digestion. Cell nuclei were labeled with TOTO3-iodide (Invitrogen, 1:5000). Embryos were mounted in 1% agarose in 80% glycerol/PBS solution and imaged on a Leica SP8 confocal microscope.

### Kaede Photoconversion

*Tg(lhx5:Kaede)^b1204^* larvae were anesthetized and mounted laterally in 1.2% low melting point agarose. A small number of cells expressing Kaede were photoconverted from green to red (Ando et al., 2002) using the 405 nm laser line on a Leica SP8 confocal microscope (objective 25×/0.95NA) by selecting a region of interest (ROI). Mounting the larvae laterally ensured that stray light away from the focal plane would only photoconvert the habenular neuropil. Images of the initial photoconverted region were taken. For both tract-tracing and fate map purposes, larvae were removed from the agarose and incubated for 24 h post conversion, then anesthetized in 0.2% tricaine (MS222, Sigma), placed into Ringer’s solution with tricaine, enucleated and embedded in 0.8% low melt agarose with fish water and imaged on a Leica SP8 confocal microscope.

### Image Analysis and Editing

Confocal stacks were processed using Fiji (ImageJ) and/or Volocity and/or Imaris. Cell size was measured on confocal images using Fiji. In some cases, digital images were inverted and converted into 8 bit gray scale, such that DiI labeled cells and fibers appear black on a white ground. Images and figures were assembled using Adobe Photoshop.

## Results

### Connections of the Habenula in Adult Zebrafish Visualized by Direct DiI Application to the Left and Right Habenulae

**Figure 1:**
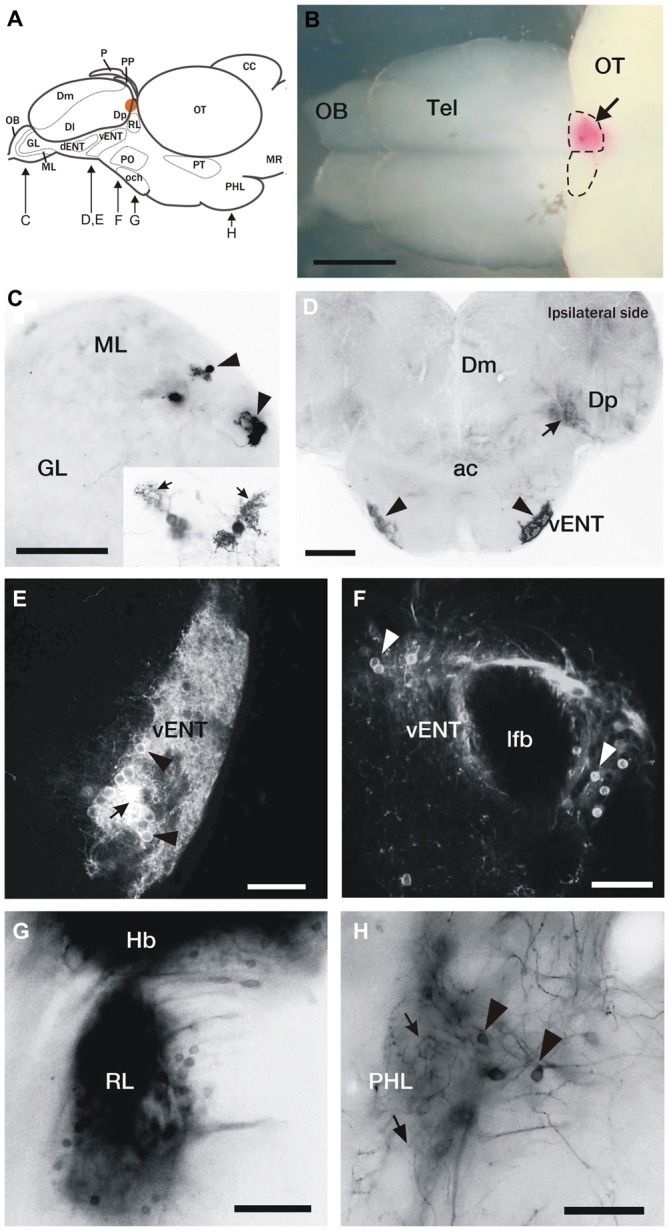
**DiI labeling of connections of the habenula in adult zebrafish. (A)** Brain schematic showing the levels of sections in **(C–H)**. Images are dorsal view of whole brain with anterior to the left **(B)** or transverse sections **(C–H)** through the brain of adult zebrafish after DiI application to the left or right habenula (orange area in **A** and pink in **B**). **(B)** Location of a typical DiI application into the right habenula (arrow). Dotted lines mark the limits of both habenulae. **(C)** Labeled mitral cells located in the dorsomedial region of the mitral cell layer in the olfactory bulb (arrowheads). Level of this section corresponds to Figure 2A. **Inset:** Detail of two mitral cells, showing characteristic dendritic arbors (arrows). **(D)** Labeled cells located bilaterally in the ventrolateral telencephalon (vENT, arrowheads) and neuropil formed by mitral cell collaterals in Dp (arrow). Note that although the vENT is labeled bilaterally, the ipsilateral nucleus (right) is more intensely labeled. Level of this section corresponds to Figure 2C. **(E,F)** Confocal images of the cells located in the vENT. At rostral levels **(E)**, small neuronal cell bodies (arrowheads) are densely distributed among a dense network of cell processes (arrow; this level of the nucleus corresponds to Figure 2C). At caudal levels **(F)**, more sparsely distributed neurons (arrowheads) surround the lateral forebrain bundle (this level of the nucleus corresponds to Figure 2D). **(G)** Labeled cells in a nucleus located in the prethalamus that may be the rostrolateral nucleus (this section corresponds to level represented in Figure 2E). **(H)** Labeled neurons (arrowheads) and beaded axons (arrows) located in the posterior hypothalamic lobe (this section corresponds to level shown Figure 2G). Scale bars: 500 μm in **(B)**, 50 μm in **(C,E,F,H)** and 100 μm in **(D,G)**.

**Figure 2:**
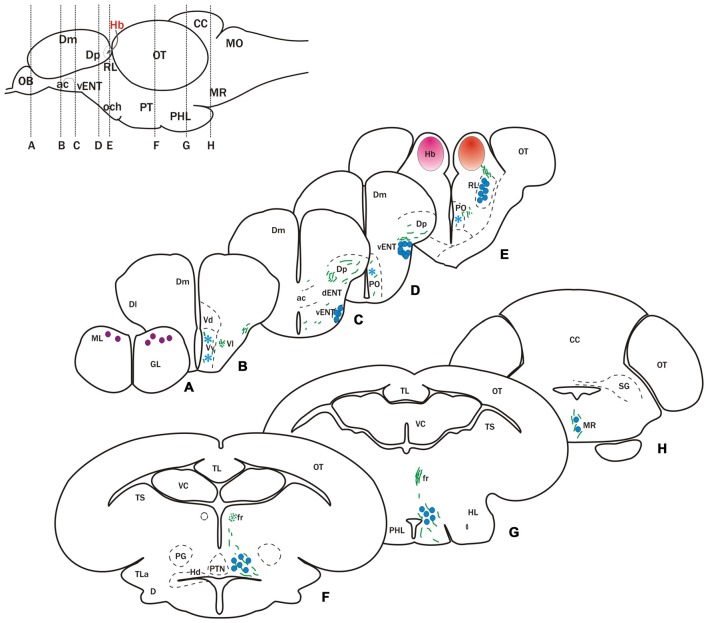
**Cells and fibers labeled after DiI application to the habenula.** Schematic at the top left shows a lateral view of the brain indicating position of transverse sections shown in **(A–H)**.** (A–H)** Schematic representation of transverse sections showing labeled fibers (green lines) and neurons (circles and asterisks) after DiI application to the left (pink circle in **E**) or right (red circle in **E**) habenula. Blue circles denote cells that project bilaterally to both habenulae, while purple circles indicate cells project only to the right habenula. Asterisks indicate occasionally labeled cells. Parapineal cells, which project to the left habenula, are not represented.

Lipophilic DiI tracing was used to identify the main afferents to the habenulae in the adult zebrafish brain. This was achieved by DiI application to either the left or right habenula (Figures 1A,B, 2E) followed by a period of time to allow the dye to diffuse along the plasma membranes of axons and cell bodies. As the habenulae are highly asymmetric, we noted which habenula was labeled and annotated the data accordingly. However, DiI will not only be incorporated by terminal fields in the habenula, but also by fibers traveling through the application point. Thus interpretation of asymmetries in projections based on direct DiI application to the habenula should be taken with caution. Parapineal or pineal afferents to the habenula were not studied, as both were removed during dissection of the brain.

#### Telencephalic Afferents to the Habenula

In two of 15 experiments, DiI bilaterally labeled mitral cells in the dorsomedial region of the olfactory bulb (Figures 1C, 2A) consistent with previous descriptions (Miyasaka et al., 2009, 2014; Gayoso et al., 2011, 2012). In a few cases (4 out of 15 experiments), we also observed labeled fibers in Dp (Figures 1D, 2C,D). These Dp fibers most probably correspond to collaterals of mitral cell axons projecting to the right habenula (see below), as described in the larva by Miyasaka et al. (2009). Given previous publications (Miyasaka et al., 2009, 2014), the inconsistent labeling of retrogradely labeled mitral cells was probably due to insufficient time for diffusion of DiI to always label the cells.

In all cases of DiI application to the left or right habenula, we observed bilaterally labeled cells in a compact, rostrocaudally elongated nucleus in the ventrolateral telencephalon (Figures 1D–F, 2C,D). Although this nucleus is bilaterally labeled, we consistently observed a higher number of labeled cells ipsilateral to the side of DiI application. The nucleus extends in the subpallium from the level of the anterior commissure (ac) rostro-ventrally (Figures 1D,E, 2C) to the telencephalic peduncle (the stalk of tissue that connects the telencephalon and the diencephalon, also called “impar telencephalon”) caudo-dorsally (Figures 1F, 2D). Labeled cells in this nucleus had rounded cell bodies, were small (average size 8.13 ± 0.61 μm; *n* = 8 cells), intensely stained and densely packed (Figures 1E,F). Based on its location and connections, this nucleus appears to correspond with the habenulopetal entopeduncular nucleus (ENT) reported in trout (Yáñez and Anadón, 1996) and goldfish (Villani et al., 1996) and to the vENT previously described in zebrafish (Wullimann et al., 1996; Amo et al., 2014).

In addition to the habenula afferents described above, occasionally we observed few labeled scattered cells in the ventral area of the subpallium (Vv; 3 out of 15 cases) and in the parvocellular preoptic nucleus (1 out of 15 cases; Figures 2B,D,E).

#### Habenula Afferents from Outside the Telencephalon

In all experiments, DiI application to either right or left habenula bilaterally labeled cells in a nucleus in the rostral diencephalon, located just ventral to the habenula and lateral to the ventrolateral thalamic nucleus (Figures 1G, 2E). Based on location, these cells are probably in the rostrolateral nucleus (RL) described in zebrafish and other teleosts (Saidel and Butler, 1997). It should be noted, however, that because of the proximity to the habenula, spread of dye from the labeling site could not be completely ruled out.

After DiI application to either the right or left habenula (in 6 out of 15 experiments), numerous cells were labeled in a region of the posterior tuberculum located between the preglomerular complex and the posterior tuberal nucleus (Figure 2F). These cells were quite scattered, as opposed to being in a compact nucleus, and formed a stream extending caudally into the posterior hypothalamic lobe (Figures 1H, 2G). They were labeled bilaterally following DiI applications to either the right or left habenula. In the vicinity of the labeled cells, we also observed some beaded axons that reached the dorsal zone of the periventricular hypothalamus (Figure 2F) and the hypothalamic posterior lobe (Figures 1H, 2G). Although the exact origin of these axons could not be assessed, reciprocal tracing experiments (see below) suggest that at least some of them could originate from habenula cells.

Finally, at rostral rhombencephalic regions, a few cells were observed in the median raphe (3 out of 15 experiments; Figure 2H).

DiI tract-tracing experiments also allowed us to examine habenular efferents and our observations are in agreement with results reported by other studies (Aizawa et al., 2005; Gamse et al., 2005; Bianco et al., 2008; Amo et al., 2010, 2014), and show dorsoventral differences in the innervation of the interpeduncular nucleus (IPN) from left and right habenula and bilateral innervation of the median raphe.

### Connections of the Habenula in Adult Zebrafish Visualized by Reciprocal DiI Experiments and Transgenic Lines

In order to study the distribution of the terminal fields in the habenula and get a better understanding of the asymmetry of projections, we targeted the regions described above for reciprocal DiI application to anterogradely label projections. This approach also allowed us to assess if any of the putative afferent neurons had been inadvertently labeled due to their axons passing through the habenular commissure without terminating in the habenulae.

#### Olfactory Bulb

**Figure 3:**
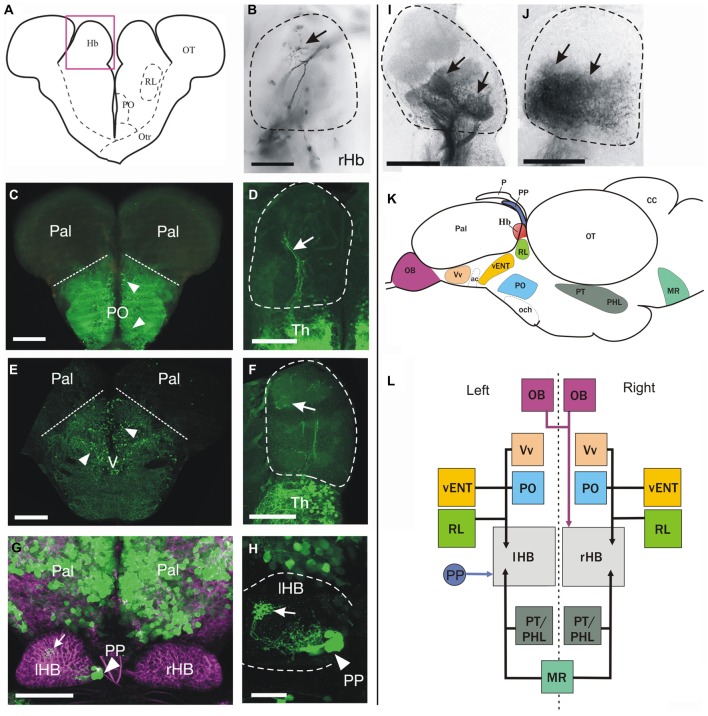
**Cell populations projecting to the habenula identified by anterograde DiI labeling and/or transgene expression. (A–J)** Schematic of a transverse section on the brain at the level of the habenulae **(A)**. The pink box approximates the area shown in **(B,D,F,I,J)**. Images are from transverse sections **(B–F,I–J)** or dorsal views with anterior to the top **(G,H)** of zebrafish brains. Dotted lines in **(B,D,F,H–J)** mark the limits of the habenula. Images are of adult brains unless otherwise stated.** (B)** Labeled fibers (arrow) dorsally in the right habenula after DiI application in the olfactory bulb.** (C)** GFP+ neurons (arrowheads) in preoptic areas of a *Tg(isl1:GFP)^rw0^* fish. Dotted lines mark the approximate limit between preoptic areas (ventral) and pallium (dorsal).** (D)** Sparse GFP+ fibers in the right habenula (arrow) of an adult *Tg(isl1:GFP)*^rw0^ fish. **(E)** GFP+ neurons (arrowheads) in the subpallium of a *Tg(1.4dlx5a-dlx6a:GFP)*^ot1^ fish. Dotted line marks the approximate pallial-subpallial boundary.** (F)** Very few GFP+ fibers (arrow) in the habenula of a *Tg(1.4dlx5a-dlx6a:GFP)*^ot1^ fish.** (G)** GFP expression (green) in 1 month old (1mpf) *Et(gata2:GFP)*^bi105^ juvenile zebrafish counter stained against tubulin (magenta) showing GFP+ neurons in the pallium and parapineal (PP, arrowhead). Note that the parapineal sends axons that terminate in the left habenula (arrow). Image is a dorsal view, anterior to the top. **(H)** High magnification image of the left habenula shown in **G** (after subtracting tubulin channel) showing that GFP+ fibers in the habenula (arrow) originate from the parapineal (PP, arrowhead). **(I)** Confocal image of the habenula showing labeled fibers (arrows) in the habenula after DiI application to the rostral vENT. Although more intense labeling is observed in ventral regions of the habenula, precise habenula subnuclei could be delineated using this method. **(J)** Labeled fibers (arrows) in the ventral half of the habenula after DiI application to the posterior hypothalamic lobe. **(K,L)** Schematics showing a summary of the main afferent areas to the habenula (**K**: outline of the brain in lateral view, **L**: boxes diagram). **(K)** The brain nuclei that project to the habenula are shown as colored areas. **(L)** Boxes represent main nuclei projecting to the habenula, while arrows represent projections. Asymmetric projection from the parapineal (PP) to left habenula (lHB) is shown with a blue arrow. Asymmetric projection from mitral cells of the olfactory bulbs (OB) to right habenula (rHB) is shown with a pink arrow. All other afferent areas that seem to innervate both left and right habenula are represented with black arrows. Scale bars: 150 μm in **(B–F,I–J)**; 100 μm in **(G)**, 50 μm in **(H)**.

DiI application to the olfactory bulb (*n* = 2) led to labeling of the lateral and medial olfactory tracts, and a terminal field in Dp (not shown). A thin tract reached the habenula and decussated in the habenular commissure. Regardless of whether DiI was applied to the right or left olfactory bulb, there was labeling in a small terminal field of the right dorsal habenula (Figures 3A,B). These results are in agreement with the telencephalic olfactory targets reported in previous studies in the zebrafish adult (e.g., Gayoso et al., 2011, 2012) and larvae (Miyasaka et al., 2009). DiI application to the olfactory bulbs was performed using a dorsal approach only.

#### Subpallium, Pallium and Preoptic Areas

Previous literature in trout, larval zebrafish and goldfish (Villani et al., 1996; Yáñez and Anadón, 1996; Hendricks and Jesuthasan, 2007) has described afferents to the habenula from the subpallium, pallium and preoptic region. Although we did see occasionally a few scattered cells in the subpallium and preoptic region after DiI application to the habenula in zebrafish (see above), we did not observe any pallial cells labeled in any experiment. Given the disparity between our data and the published record, we used an alternative approach to assess if pallial neurons project to the habenula through analysis of the transgenic line *Et(gata2:GFP)^bi105^*. In addition, we also analyzed two transgenic lines in which large numbers of subpallial and preoptic cells (among other populations) express GFP: *Tg(1.4dlx5a-dlx6a:GFP)*^ot1^ and *Tg(isl1:GFP)*^rw0^.

Numerous GFP+ neurons are present in the subpallium and preoptic region (Figure 3C) of adult *Tg(isl1:GFP)^rw0^* transgenic fish. A few GFP+ fibers of unknown origin reached the ventrolateral region of both habenulae forming a sparse terminal field (Figure 3D). Likewise, adults carrying the *Tg(1.4dlx5a-dlx6a:GFP)*^ot1^ transgene have a large number of GFP+ cells (mostly GABAergic neurons; Yu et al., 2011) in ventral (Vv), supracommissural (Vs), dorsal (Vd) and posterior (Vp) regions of the subpallium (Figure 3E), the preoptic area, and prethalamus. However, despite this broad expression of the transgene, we observe very few GFP+ fibers in the habenula (Figure 3F). In addition, *Et(gata2:GFP)*^bi105^ transgenic fish expresses GFP in numerous cells located in the pallium (Figure 3G), excluding the olfactory bulb (see Folgueira et al., 2012), but have no GFP+ fibers of pallial origin in the habenulae (Figures 3G,H). In consequence, analysis of transgenic lines did not identify any major pallial projections or additional subpallial and preoptic projections to the habenula. Thus these analyses support the tracing data and fail to identify a significant direct projection from the pallium to the habenulae.

#### Ventral Entopeduncular Nucleus

DiI labeling of the rostral vENT (*n* = 2) led to strong labeling of fiber bundles in the tract of the habenular commissure that innervate the ipsilateral habenula (Figure 3I). Some fibers also decussated in the habenular commissure to innervate the contralateral side. Labeled terminals were mainly found in the ventral half of the habenula, confirming the projection from vENT to ventral habenula previously reported by Amo et al. (2014), although we also saw some fibers invading the dorsal half of the habenulae. No labeled cell bodies were observed in the habenula, strongly suggesting that the connection between the vENT and the habenula is not reciprocal.

#### Parapineal

Owing to the difficulty of DiI application to the parapineal without affecting other structures, we used the *Et(gata2:GFP)^bi105^* transgenic line (Folgueira et al., 2012) to study the distribution of parapineal afferents to the habenula in juvenile zebrafish. We observed a short and compact GFP+ tract of parapineal axons entering the left habenula exclusively, its fibers forming profuse terminal fields extending from medial to lateral (Figures 3G,H). These results confirm the asymmetric innervation pattern of the habenula by the parapineal reported in early developing zebrafish (Concha et al., 2003).

#### Posterior Hypothalamic Lobe

Finally, DiI application to the posterior hypothalamic lobe (*n* = 1) led to conspicuous labeling of a dense neuropil in the ventral half of both habenulae (Figure 3J). In addition, we also saw a few labeled cells among the neuropil in the habenula (Figure 3J), indicating that the connection between the habenula and hypothalamus may be reciprocal.

Taken together, our results suggest that the main projection from the telencephalic lobes to the habenula comes from the vENT nucleus (located in the ventrolateral telencephalon), but not from other subpallial, preoptic or pallial domains (see Figures 3K,L).

### GFP+ vENT Neurons in *Tg(lhx5:GFP)*^b1205^ Adult Zebrafish Project to the Habenula

Two distinct entopeduncular nuclei have been described in the adult zebrafish: the dorsal and ventral entopeduncular nuclei (dENT and vENT; see Wullimann et al., 1996). This is unlike other teleosts, where a single nucleus has been reported (Villani et al., 1996; Yáñez and Anadón, 1996). In order to characterize further the structures previously termed dENT and vENT in zebrafish, we investigated their location, neurochemistry and connectivity.

**Figure 4:**
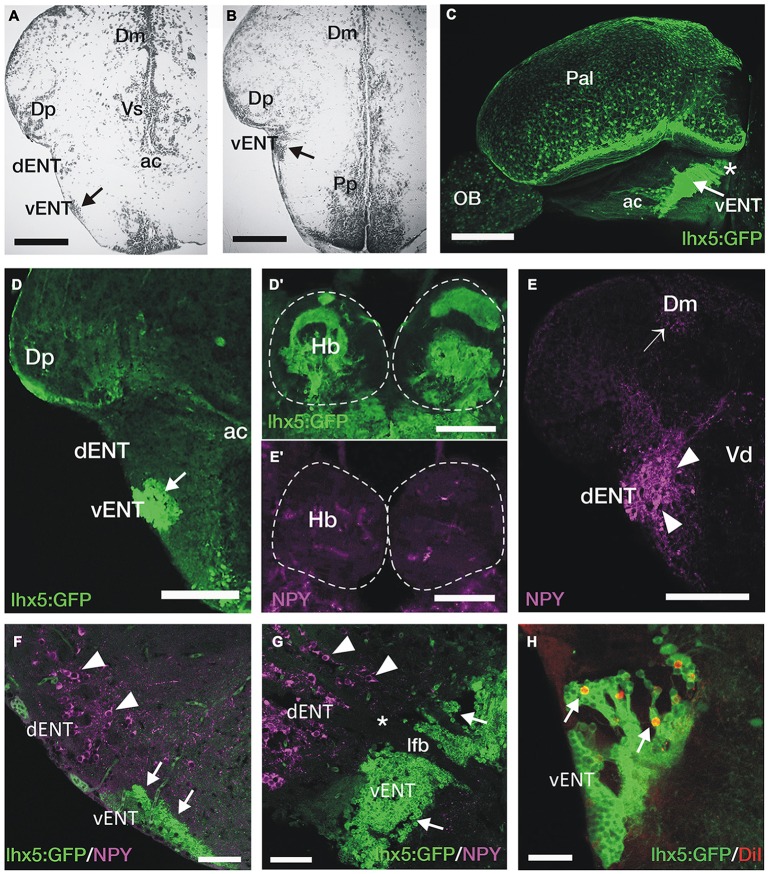
**GFP+ vENT neurons and habenular innervation in *Tg(lhx5:GFP)*^b1205^ transgenic zebrafish.** Images are transverse sections **(A,B,D–F,H)**, lateral view **(C)** or parasagittal section **(G)** of adult zebrafish wild type **(A,B,E,E′)** or *Tg(lhx5:GFP)*^b1205^ **(C–D′,F–H)** brains. **(A,B)** Nissl stained sections showing the cytoarchitecture of the vENT (arrows in **A** and **B**) and dENT at anterior (same level as shown in schematic Figure 2C) and posterior (same level as shown in schematic Figure 2D). By following serial sections it can be noted that only the vENT extends into caudal areas of the telencephalon (arrow in **B**). **(C)** Lateral view of intact brain from a *Tg(lhx5:GFP)*^b1205^ fish (anterior to the left) showing GFP+ cells in the vENT (arrow), among other structures and areas. Note that vENT cells form a band extending from the level of the anterior commissure (ac) into the telencephalic peduncule (indicated with a star). **(D)** GFP+ cells in the vENT (arrow), but not in the dENT, of a *Tg(lhx5:GFP)*^b1205^ fish. **(D′)** Dense neuropil of GFP+ fibers in the habenula of a *Tg(lhx5:GFP)*^b1205^ fish. Dotted lines mark the habenulae. **(E)** NPY+ cells in the dENT and fibers (arrowheads) in the medial area of the pallium (Dm; arrow). **(E′)** Very few NPY+ fibers are found in the habenulae. Note that some of the labeling observed in the image is caused by autofluorescence of blood vessels. **(F,G)** Double immunohistochemistry against NPY and GFP in transverse **(F)** and parasagittal (**G**; anterior to the left) sections of a brain from a *Tg(lhx5:GFP)*^b1205^ fish. Note that NPY+ cells are restricted to the dENT (arrowheads). GFP+ cells were observed in the vENT (arrows). Note in **(G)** that the dENT (arrowheads) does not extend as far caudal as the vENT (arrows) and there is a clear gap (star) between the two nuclei. Bundles of axons from the lateral forebrain bundle (lfb) travel through both nuclei. **(H)** Retrogradely DiI labeled cells (arrows) in the vENT of a *Tg(lhx5:GFP)*^b1205^ fish after tracer application to the habenula. Scale bars: 150 μm in **(A,B,D–E′)**; 250 μm in **(C)**; 100 μm in **(F,G)** and 50 μm in **(H)**.

The vENT is the only one of these two nuclei that extends from the ac to the telencephalic peduncle (Figures 4A,B). In comparison with the dENT, the vENT is formed of smaller and more densely packed neurons (Figures 2D, 4A,B). Labeled cells in the ventrolateral telencephalon after DiI application to the habenula also showed this location and extension, indicating that the habenula projecting nucleus is the vENT (Figures 2D, 4B). Amongst expression in other areas of the brain, we identified GFP-positive (GFP+) cells in the entopeduncular region of *Tg(lhx5:GFP)*^b1205^ transgenic fish (Figure 4C). In this line we found closely packed GFP+ cells in the vENT (Figures 4C,D) and a dense GFP+ neuropil in both right and left habenulae (Figure 4D′), whereas the dENT lacked GFP expression (Figure 4D). This indicated that this transgenic line could be employed to differentiate between vENT and dENT nuclei.

Previous anatomical studies (Mueller and Guo, 2009; Ganz et al., 2011) have proposed that the dENT projects to the habenula and should be considered “the entopeduncular nucleus proper”, with the vENT being referred to as the “bed nucleus of the stria medularis”. Our results do not support these homologies, as tract-tracing experiments show a major projection to the habenula from the vENT, in agreement with results of Amo et al. (2014), but no afferents from the dENT. We decided to undertake some further experiments to assess the validity of this conclusion.

Castro et al. (2006) have shown that the dENT contains NPY-positive (NPY+) neurons. We therefore used immunohistochemistry to see if there were NPY+ projections to the habenulae that might originate from the dENT. We confirmed that there are NPY+ cell bodies in the dENT and, among other areas, very high density of NPY+ processes innervating parts of the pallium (Dm, Figure 4E; Castro et al., 2006). Indeed DiI application to rostral Dm labeled cells in the dENT, but not in the vENT (not shown). We observed almost no NPY+ fibers in the habenulae (Figure 4E′). In addition, double immunostaining against NPY and GFP in *Tg(lhx5:GFP)*^b1205^ adult zebrafish showed that vENT GFP+ cells are NPY negative (Figure 4F). Moreover, NPY+ cells of the dENT do not extend caudally into the telencephalic peduncle, unlike vENT cells (Figure 4G). Parasagittal brain sections also revealed a separation between the NPY+ dENT population and the GFP expressing vENT (Figure 4G). After DiI application to the habenula of *Tg(lhx5:GFP)*^b1205^ fish, we observed double GFP/DiI labeled cells in the vENT (Figure 4H). No DiI labeled cells were observed in the ventrolateral telencephalon outside of the GFP+ vENT region.

These results demonstrate that axons originating from vENT neurons, and not the dENT, project to the habenula. In addition, they show the dENT contains NPY+ neurons that are likely to project to the dorsal telencephalon but not to the habenulae.

Taken together, our analyses on the adult zebrafish brain provide strong evidence that the vENT and the olfactory bulb provide the principal telencephalic inputs to the habenulae. As the *Tg(lhx5:GFP)*^b1205^ transgene labels the vENT- habenula system, we next used this transgene and the related Kaede allele [*Tg(lhx5:Kaede)*^b1204^, (Gao et al., 2012; Zhang et al., 2012)] to investigate the development of the vENT.

### GFP+ Neurons in *Tg(lhx5:GFP)*^b1205^ Embryos/Larvae Innervate the Habenulae as in Adults

The developmental origins of the vENT and dENT are a matter of some controversy and debate (Wullimann and Mueller, 2004; Mueller and Guo, 2009; Ganz et al., 2011). Consequently, we decided to investigate the developmental origins of these regions, particularly because the vENT appears to be the main source of afferents to the habenula in the adult. First, we analyzed if there is GFP expression in the vENT of larval *Tg(lhx5:GFP)*^b1205^ fish, as observed in the adult.

**Figure 5:**
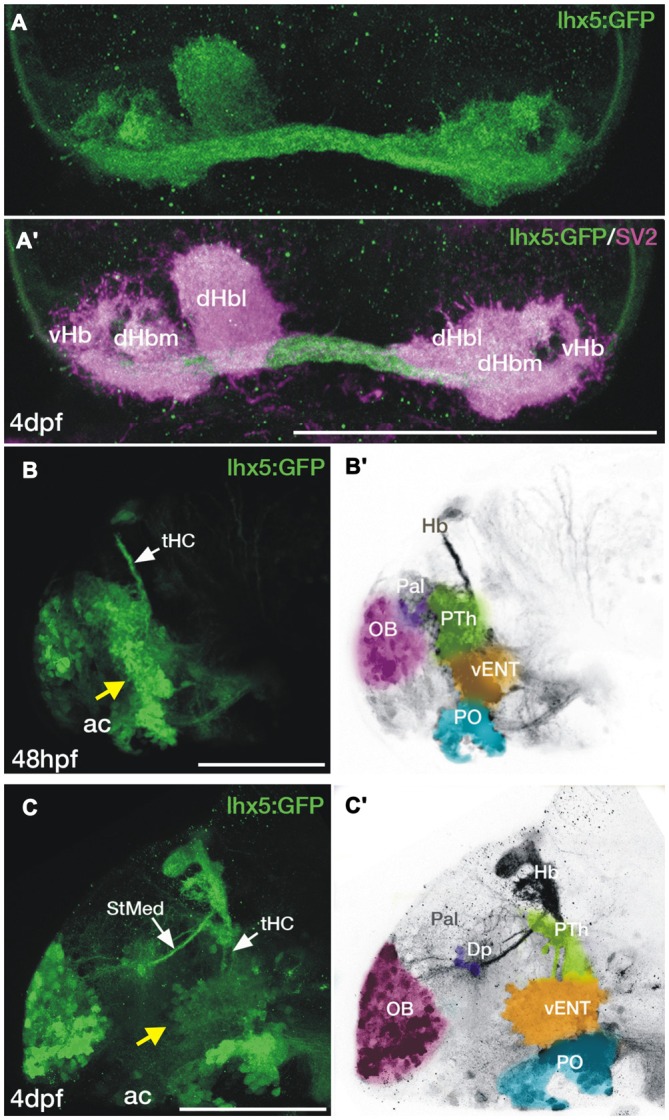
**Transgene expression in the forebrain of *Tg(lhx5:GFP)*^b1205^ embryos and larvae. (A,A′)** Dorsal views of the habenula of a 4dpf *Tg(lhx5:GFP)*^b1205^ fish labeled with anti-GFP only **(A)** or anti-GFP (green) and anti-SV2 (magenta) antibodies **(A′)**. GFP+ axon terminals (green) innervate all habenula neuropil domains visualized with SV2 (magenta). **(B–C′)** Lateral views of the forebrain of a *Tg(lhx5:GFP)*^b1205^ fish at 48 hpf **(B,B′)** and 4dpf **(C,C′)**. **(B)** GFP+ cells and processes in 48 hpf *Tg(lhx5:GFP)*^b1205^. Note that the tract of the habenular commissure is labeled at this stage (tHC, white arrow). Yellow arrow points to putative prethalamic eminence/vENT. **(B′)** Image shown in **(B)** has been converted to gray scale and false colored areas show the different presumptive domains. GFP+ cells and processes form a continuous band at the telencephalic-diencephalic boundary **(B)**, extending from presumptive prethalamus (PTh, labeled in green), through the putative prethalamic eminence/vENT (vENT, labeled in yellow) and preoptic regions (PO, labeled in blue). A number of GFP+ neurons are also present in the olfactory bulb (OB, labeled in pink). **(C)** GFP+ cells and processes in 4dpf *Tg(lhx5:GFP)*^b1205^ fish. Note that at this stage the main afferent tracts to the habenula, the stria medullaris (StMed) and the tract of the habenular commissure (tHC) are visible. Yellow arrow points to putative prethalamic eminence/ vENT. **(C′)** Image shown in **(C)** has been converted to gray scale and false colored areas show the different presumptive domains. There are a number of GFP+ cells rostrally in the OB (pink area), a few GFP+ cells and fibers in Dp (purple). Prethalamus, vENT and preoptic regions are again labeled in green, yellow and blue respectively. Scale bars: All 100 μm.

By 4dpf, the habenulae of *Tg(lhx5:GFP)*^b1205^ larvae show a dense GFP+ neuropil throughout the various habenular sub-nuclei (Figures 5A,A′), indicating that the transgene must label areas afferent to the habenulae from early stages. Indeed, in support of this, main afferent tracts to the habenulae, the *stria medullaris* and the *tract of the habenular commissure* are labeled (Figures 5B–C). At 2dpf, we observe many GFP+ mitral cells in the olfactory bulb and a thick band of GFP+ neurons and processes at the caudal telencephalon and around the telencephalic-diencephalic boundary (Figure 5B). By 4dpf, there is GFP+ expression in mitral cells, a few neurons located in the ventrolateral dorsal telencephalon (likely in Dp) and in an adjacent neuropil (Figure 4C). At this stage and comparing with 2dpf, the GFP+ band of cells at around the telencephalic-diencephalic boundary has extended rostrocaudally and the different domains are easier to identify. The caudal boundary of this band of GFP+ cells is located anterior to the *zona limitans intrathalamica*, as determined by comparison with expression of the *(−2.7shh:GFP)*^t10^ transgene (data not shown). Consequently, dorsally, GFP expression is likely located within derivatives of the prethalamus and prethalamic eminence (Figure 5C; the prethalamic eminence has also been named as thalamic eminence or eminentia thalami; see Wullimann and Mueller, 2004; Puelles, 2007; Mueller and Guo, 2009). Previous literature has suggested that the vENT originates from the prethalamic eminence (Mueller and Guo, 2009; Ganz et al., 2011). From 2 to 4 dpf, we did observe that some GFP+ cells extend from the putative prethalamic eminence (yellow arrow in Figure 5B) into the telencephalic lobes (yellow arrow in Figure 5C). We tentatively identified these cells as presumptive larval vENT (Figures 5B′,C′). GFP+ expression ventral to this domain is likely to be located within preoptic regions (Figures 5B′,C′).

### Identification of the Larval vENT and Other Habenulopetal Nuclei Using *Tg(lhx5:Kaede)*^b1204^ Transgenic Fish

**Figure 6:**
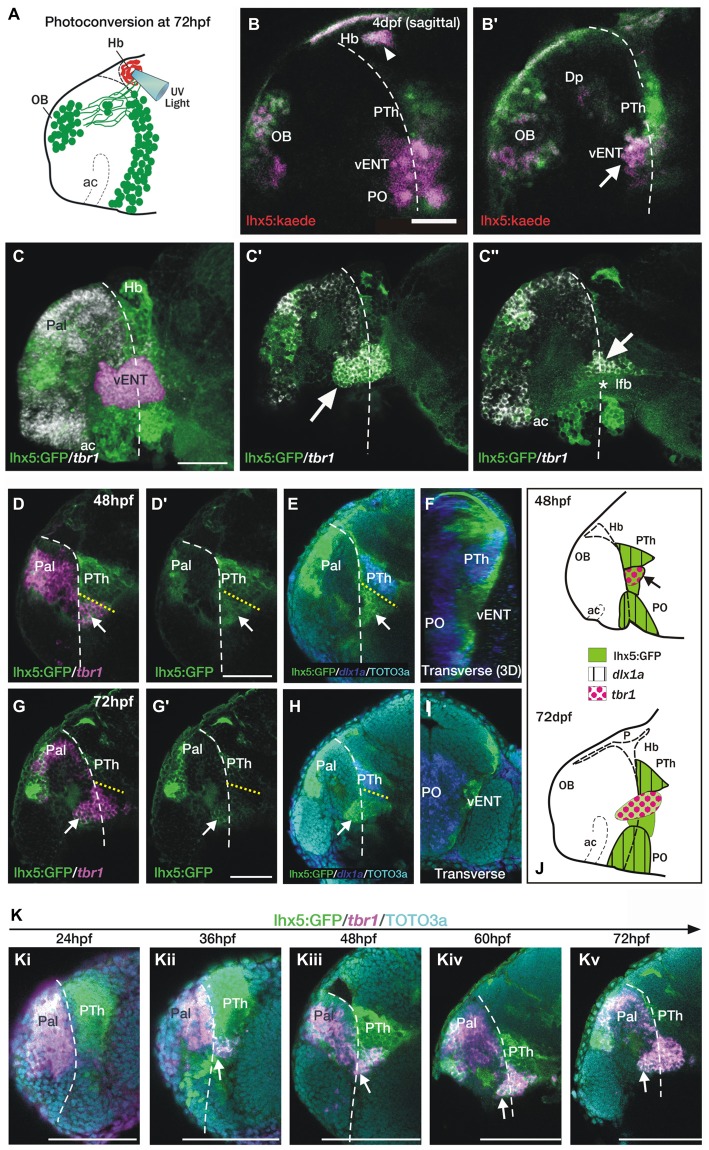
**The telencephalic vENT is a major source of habenula afferents, is diencephalic in origin and originates from the prethalamic eminence.** *The vENT projects to the habenula.* **(A)** Schematic summarizing experimental setup. Habenula neuropil of 72 hpf *Tg(lhx5:Kaede)*^b1204^ was photoconverted with a cone of UV light. Photoconversion was made with a lateral approach in order to avoid conversion of areas ventral to the habenula. **(B,B′)** Lateral views of single *Z*-slices of *Tg (lhx5:Kaede)*^b1204^ larvae (**B′** is closer to the midline) imaged at 4dpf after photoconversion of the habenula neuropil at 72 hpf. Dotted line marks the anterior intraencephalic sulcus. Some red Kaede cells (magenta in the figure) are observed in the olfactory bulb (OB) and preoptic region (PO), and a few fibers in Dp. A number of red Kaede cells were observed in the domain identified as presumptive larval vENT (arrow in **B′**). Note red Kaede fibers at the photoconversion area in the habenula (arrowhead in **B**). *The vENT expresses tbr1 and straddles the telencephalic/diencephalic boundary at 4dpf.*** (C–C″)** Lateral views of *Tg(lhx5:GFP)*^b1205^ inmunostaining against GFP (green) and FISH against *tbr1* (grayscale). In **C**, the vENT has been false colored in pink. Dotted lines on lateral views mark the anterior intraencephalic sulcus, which runs from the optic recess at the base of the optic stalks ventrally to the pallial/diencephalic boundary dorsally. **(C)** 3D projection of a confocal stack showing *tbr1* and GFP co-expression in the putative vENT (false colored in pink to differentiate from pallial and subpallial *tbr1a* expression). **(C′,C″)** Single confocal *z*-slices at lateral **(C′)** and medial levels **(C″)** showing GFP and *tbr1* coexpression in vENT neurons (arrow). Note that the vENT extends from the diencephalon into the telencephalic lobes (dotted line marks the anterior intraencephalic sulcus). At medial levels, the larval vENT surrounds the lateral forebrain bundle (lfb, star in **C″**), as has been observed in the adult (see Figure 1F). *The vENT is diencephalic in origin and originates from the prethalamic eminence.* Lateral **(D,E,G,H)** and transverse views **(F,I)** of Tg(*lhx5:GFP)*^b1205^ fish labeled with anti-GFP (green) and FISH for *tbr1* (pink) **(D–D′,G,G′)** or *dlx1a* (blue) **(E,F,H,I)** at 48 hpf **(D–F)** and 72 hpf **(G–I)**. All images are single confocal z-slices taken from volumes, anterior to the left. **(D–F)** Lateral **(D,E)** and transverse view after 3D rendering **(F)** of Tg(*lhx5:GFP)*^b1205^ 48 hpf larva labeled with anti-GFP (green) and FISH for *tbr1*+ (pink in **D**) and *dlx1a*+ (blue in **E**,**F**). Dotted yellow line in **(D,E)** marks the dorsal limit of the *tbr1*+ domain. **(D)** A group of GFP and *tbr1*+ cells (arrow) are situated in the diencephalon, just caudal to the telencephalic/diencephalic boundary. These cells were identified as prethalamic eminence/prospective vENT. **(D′)** Same image as **(D)** but only showing GFP expression (green channel). Arrow points to prethalamic eminence/prospective vENT. **(E)** GFP and *dlx1a+* cells in the prethalamus, just dorsal to the prethalamic eminence/prospective vENT (arrow). **(F)** Transverse section shows the relative mediolateral position of the prethalamus, vENT and preoptic areas. **(G–I)** Lateral **(G,H)** and transverse section **(I)** of Tg(*lhx5:GFP)*^b1205^ 72 hpf larva labeled with anti-GFP (green) and FISH for *tbr1*+ (pink in **G**) and *dlx1a*+ (blue in **H** and **I**). Dotted yellow line in **(D,E)** marks the dorsal limit of the *tbr1*+ domain. **(G)** The GFP+ vENT expresses *tbr1* extends from the ventrolateral telencephalon to the rostral diencephalon (arrow points to the rostral portion of the vENT, dotted yellow line marks the dorsal limit of the *tbr1+* domain). **(G′)** Image shown in **(G)** showing just GFP expression (green channel). **(H)** *dlx1a+* domain in the prethalamus (PTh), just dorsal to the diencephalic portion of the vENT (arrow points to rostral portion of the vENT, dotted yellow line marks the dorsal limit of the *tbr1+* domain in the diencephalon). **(I)** Transverse section shows the GFP+ vENT (green) laterally located and *dlx1a+* PO (blue) areas more medially. **(J)** Schematic representations of the brain of 48 and 72 hpf larvae showing expression of lhx5:GFP, *dlx1a* and *tbr1* in the prethalamus (PTh), prethalamic eminence (arrow) and preoptic region (PO). *Time-series showing the development of the prethalamic eminence that gives rise to the vENT.* **(K)** Time series showing *tbr1* expression from 24 to 72 hpf in Tg(*lhx5:GFP)*^b1205^. **(i)** At 24 hpf, there is no *tbr1* expression in rostral diencephalon; **(ii)** At 36 hpf brain, *tbr1* expression is observed in a subset of the GFP+ cells in the diencephalon (arrow) close to the the telencephalic/diencephalic boundary (marked by dotted line). These cells are likely to be within the prethalamic eminence/prospective vENT; **(iii–v)** From 48–72 hpf, vENT cells coexpressing *tbr1* and GFP extend into the telencephalic lobes (arrow points to rostral portion of the vENT). Scale bars: **(B,C,D′,G′)** 50 μm; **(K)** 100 μm.

To map the developing brain regions projecting axons to the habenula in the larva, we focally photoconverted the habenular neuropil of 72 hpf *Tg(lhx5:Kaede)*^b1204^ embryos from green to red (Figure 6A). The labeling procedure also invariably led to photoconversion of Kaede protein in axons of the habenular commissure*.* After 24 h, in some individuals red Kaede protein spread retrogradely to label mitral cells in the olfactory bulb that project to the right habenula (as well as due to the conversion of Kaede in mitral cell axons passing through the habenular commissure, *n* = 2/8; see Miyasaka et al., 2009), some individuals had a few cells in the preoptic area (*n* = 2/8) and many individuals had many cells in the domain we had tentatively identified as presumptive vENT (*n* = 6/8; Figures 6B,B′). The robust labeling of this presumptive vENT validates the supposition that it is from this domain that the neurons of the mature vENT derive. In addition, we observed red Kaede in neuropil, but not cells, of Dp [likely from mitral cells forming en-passant arbors in this region (Miyasaka et al., 2009, 2014; Figures 6B,B′)]. All these sites of labeling observed in the larva correspond to projections identified in the adult.

These results suggest that major sites of afferent neurons to the habenula establish innervation early during embryonic/larval development. In addition, they allow identification of the location of the larval vENT, which projects to the habenula from early stages and might originate from a domain of *lhx5*:GFP/Kaede identified as putative prethalamic eminence.

### Larval vENT Neurons Express *tbr1*

Ganz et al. (2011) showed that the adult vENT is *tbr1+* and may originate from the *tbr1*+ prethalamic eminence. At 4dpf, *tbr1* expression overlaps with GFP expression in the ventrolateral telencephalon of *Tg(lhx5:GFP)*^b1205^ larvae, again supporting the idea that this population of cells is the larval vENT (Figures 6C–C″). As *tbr1* shows better spatially delineated expression in the vENT than *lhx5:GFP* at this stage, we used this marker to track the likely developmental origins of this nucleus. At 48 hpf, some *tbr1+* cells are located within the diencephalon (Figures 6D,D′), sandwiched between two *dlx1a*+ domains that, posteriorly, label the prethalamus and, anteriorly, the posterior preoptic areas (Figures 6E,F,J; Wullimann and Mueller, 2004). These results confirm that the diencephalic *tbr1+* domain is located in the prethalamic eminence. The relative positions of expression domains of *tbr1* and *dlx1a* in the diencephalon are similar at later stages (Figures 6G–J).

We analyzed *tbr1* expression at different stages in order to track the development of the larval vENT (Figure 6K). At 24 hpf, *tbr1* expression was restricted to the telencephalic pallium (Figure 6Ki), but by 36 hpf a few cells expressed *tbr1* in the rostral diencephalon (Figure 6Kii). This small group of diencephalic cells is likely to be within the prethalamic eminence (Wullimann and Mueller, 2004). As development progresses, the number of *tbr1*+ cells increased and their location with respect to the intraencephalic sulcus (located between telencephalon and diencephalon) altered (Figures 6Kiii–v). Prior to 48 hpf, *tbr1*+ cells were all located caudal to the sulcus, whereas between 48 hpf and 72 hpf, cells progressively straddled the sulcus (Figures 6Kiii–v) such that by the end of this period, the rostral half of the vENT was within the telencephalic lobes. Taken together, *tbr1* and *dlx1a* expression data suggests that the vENT originates from the diencephalic prethalamic eminence, as proposed by previous studies (Mueller and Guo, 2009; Ganz et al., 2011), extending into the telencephalic lobes from 48–72 hpf.

### Fate Mapping Confirms the Diencephalic Origin of vENT Cells

**Figure 7:**
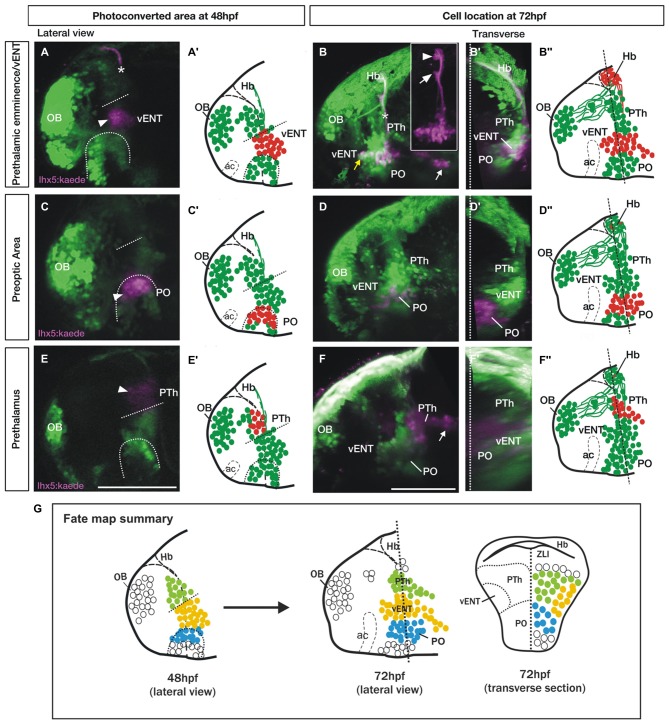
**Spatio-temporal fate map of the larval prethalamic eminence/prospective vENT and adjacent areas.** Images and accompanying schematics show Kaede photoconversion areas in lateral views of 48 hpf *Tg(lhx5:Kaede)^b1204^* fish **(A,A′,C,C′,E,E′)** and location of Kaede cells by 72 hpf in lateral view **(B–B″,D–D″,F–F″)** and transverse sections after 3D rendering **(B′,D′,F′)**. Dotted arch and lines at 48 hpf mark the dorsal limit of the preoptic area and prethalamic eminence respectively. *Photoconversion in the prethalamic eminence* (*n* = 8). **(A–B″)** Cells were photoconverted from green to red (magenta in the images) at the diencephalic prethalamic eminence of *Tg (lhx5:Kaede)*^b1204^ embryos at 48 hpf (arrowhead in **A**, red cells in schematic **A″**) and their location was then imaged by 72 hpf in lateral view (**B** and red cells in **B″**). Note in **(A)** that area of photoconversion by 48 hpf is located at the level of the tract of the habenular commissure (marked with a star). **(B′)** is transverse section after 3D rendering. Note that 24 h after photoconversion, red Kaede expressing neurons have expanded from the photoconversion area rostrally into the telencephalon (yellow arrow) and caudally into hypothalamic areas (white arrow). These red Kaede cells send afferent projections to ventral (arrow in **B** inset) and dorsal halves (arrowhead in **B** inset) of the habenula and were identified as vENT. Transverse section shows that the vENT occupies a lateral position within the forebrain **(B′)**. *Photoconversion in preoptic regions (PO)* (*n* = 9)**. (C–D″)** Cells were photoconverted in the presumed preoptic area of *Tg (lhx5:Kaede)*^b1204^ embryos at 48 hpf **(C,C′)**. Note that 24 h after photoconversion, most red Kaede cells maintain their relative rostro-caudal positions within the telencephalon and diencephalon **(D)**, and are located ventro-medially relative to the vENT when viewed in transverse section **(D′)**. Most conversions of presumptive PO (*n* = 6/9) did not result in labeling of habenula afferents. *Photoconversion of the prethalamus (PTh; n = 3)*.** (E–F″)** Cells were photoconverted in the presumed prethalamic area of *Tg (lhx5:Kaede)*^b1204^ embryos at 48 hpf **(E,E′)**. Twenty four hours after photoconversion, most red Kaede cells maintain their positions within the diencephalon and do not expand into the telencephalon **(F)**. A few red Kaede cells expand into caudal areas (white arrow). Transverse section shows that these cells occupy a dorsomedial position **(F′)**. **(G)** Summary of the results of the fate map experiments with *Tg(lhx5:Kaede)*^b1204^, with the areas of Kaede photoconversion in 48 hpf. Open circles denote cells that were not fate mapped. Level of transverse section is shown in the 72 hpf lateral view of the brain. Scale bars: All 100 μm.

In order to ascertain if the vENT does indeed originate from the prethalamic eminence, we photoconverted 48 hpf *lhx5*:Kaede+ diencephalic cells in the area we had identified as prethalamic eminence based on *dlx1a* and *tbr1* gene expression (Figures 7A,A′). The photoconverted area is adjacent to the anterior intraencephalic sulcus and ventral to the tract of the habenular commissure, so these structures have been used as anatomical landmarks (Figure 7A). After 24 h, some red Kaede cells were located quite far from the photoconversion site in the ventrolateral telencephalon (*n* = 8/8, Figures 7B–B′). These photoconverted cells could be assigned to the vENT, as they were located ventrolateraly in the telencephalon (Figure 7B′) and sent axons via the tract of the habenular commissure to form two terminal neuropils in the ipsilateral habenula (Figure 7B, arrowhead and arrow in Figure 7B inset). We also observed some red cells far from the photoconversion site and extending caudally, likely into hypothalamic regions (Figures 7B,B″). These cells could belong to any of the migratory populations reported previously in the literature (Mueller and Wullimann, 2002; Mione et al., 2008).

After photoconversion of the domain identified as preoptic region at 48 hpf (as identified by *dlx1a* expression; Figures 7C,C′; *n* = 9/9), most red cells remained localized close to the photoconversion site by 72 hpf, but a few cells extended into rostral areas (Figures 7D–D″). Transverse sections from 3D rendered brains showed that these red Kaede cells are located close to the midline, consistent with a preoptic identity (Figure 7D′). In three of nine photoconversions, a small number of red Kaede axons reached the habenula, consistent with observations in the adult and suggesting a minor preoptic-habenula projection may exist. Finally, red cells photoconverted in the prethalamus at 48 hpf (Figures 7E,E′; *n* = 3/3), either remained close to the photoconversion site or extended caudally into more posterior regions, but did not obviously invade telencephalic areas (Figures 7F–F″).

These results support the conclusions that the vENT originates from the diencephalic prethalamic eminence at early stages of development and extends into the ventrolateral telencephalon from 48 to 72 hpf (see Figure 7G for a summary of the fate map results).

### Many Larval Zebrafish vENT Neurons are Glutamatergic

**Figure 8:**
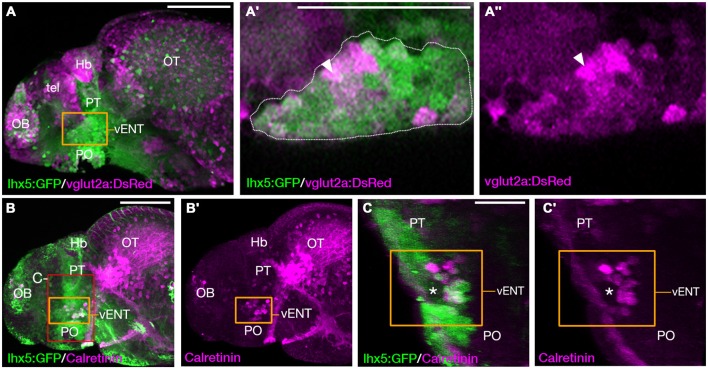
**The larval vENT contains glutamatergic and calretinin-positive populations of cells. (A,A″)** Confocal images of *Tg(lhx5:GFP)*^b1205^/*Tg(slc17a6b:DsRed)*^nns9Tg^ 4 dpf double transgenic larvae labeled with anti-GFP and anti-DsRed antibodies (green and magenta respectively). GFP expression shows the previously identified vENT (orange box in **A**, enlarged in **A′,A″**). Enlarged views of single confocal sections **(A′,A″)** show co-expression of GFP and DsRed in a subpopulation of cells (arrowheads) of the GFP+ vENT (dotted line in **A′** marks). **(B–C′)** *Tg(lhx5:GFP)*^b1205^ 4dpf larvae labeled with anti-GFP (green) and anti-calretinin (magenta). **(B,B′)** lateral view shows a subset of GFP+ vENT neurons (bound by orange box) express calretinin. **(C,C′)** 3D transverse sections of same larvae **(B)** cropped to area delineated by red box in **(B)**. Calretinin+ and GFP+ neurons within the vENT, which surrounds the lateral forebrain bundle (lfb; asterisk). Scale bars: **(A,B)** 100 μm; **(A′,C)** 50 μm.

In rodents, glutamatergic neurons within the ENT project to the lateral habenula (Shabel et al., 2012). The transgene *slc17a6b:DsRed*^nns9Tg^ (Kani et al., 2010; otherwise known as vglut2a:DsRed; Miyasaka et al., 2009) expresses DsRed in glutamatergic neurons. We observed that many *lhx5*:GFP+ cells of the vENT express the transgene and consequently are likely to contain glutamate (Figures 8A–A″). Similarly, Amo et al. (2014) reported that in the adult, vENT neurons express vglut2a mRNA and are probably glutamatergic. The calcium binding protein calretinin is also expressed in ventrolateral telencephalic areas (Castro et al., 2006) and indeed, there was considerable overlap between *lhx5:GFP* and calretinin labeling in the vENT adjacent to the lateral forebrain bundle, and ventral to the calretinin negative prethalamus (Figures 8B–C′).

## Discussion

Using tract tracing and Kaede photoconversion experiments in adult and larval zebrafish, we have revealed that the main afferent nuclei to the zebrafish habenulae are the olfactory bulb (to right habenula), subpallium, vENT, parapineal (to left habenula), nucleus rostrolateralis, preoptic area, posterior tuberculum, posterior hypothalamic lobe and median raphe.

### Zebrafish vENT is the Zebrafish Homolog of the Conserved Vertebrate ENT

Since Wullimann et al. (1996) first described the dENT and vENT in zebrafish, their homologies have been controversial. Castro et al. (2006) considered the dENT as an extension of the lateral area of the subpallium (Vl), and the vENT to be the ENT “proper”. In contrast, other authors proposed that the dENT is the ENT “proper”, while the vENT is the bed nucleus of the stria medullaris (Mueller and Guo, 2009; Ganz et al., 2011). Despite this controversy, in these studies, connectivity was not used to test the homologies of the dENT and vENT. Using tracer application to the ventral habenulae, Amo et al. (2014) found that the zebrafish vENT contains habenulopetal projecting neurons. Building on this initial finding, our reciprocal tract-tracing and Kaede photoconversion experiments confirm that afferents to the habenula in zebrafish originate only from the vENT, not the dENT. This hodological evidence confirms the vENT as the ENT “proper” in zebrafish. Accordingly, we will use this terminology (ENT) for the remainder of the article. Since the dENT does not project to the habenula, in our opinion it should be renamed to avoid confusion. Our DiI and Kaede experiments did not allow to rule in or out asymmetries in the vENT-habenula projection.

The vertebrate ENT is likely to be of diencephalic origin in all vertebrates. In zebrafish, the fate map and gene expression data above demonstrate the ENT originates from the prethalamic eminence, as delineated by *lhx5* and *tbr1* expression (Wullimann and Mueller, 2004; Mueller and Guo, 2009; Ganz et al., 2011; Lauter et al., 2013; present results). In rat, Marchand et al. (1986) found the entopeduncular nucleus is likely derived from a diencephalic domain closely associated with the ventral diencephalic sulcus. Finally, gene expression analyses show the prethalamic eminence expresses *lhx5* and *tbr1* and contributes cells to the basal telencephalon in *Xenopus*, mouse and chick (Puelles et al., 2000; Brox et al., 2004; Abellán et al., 2010) as well as in fish. Together these observations are all consistent with ENT neurons having diencephalic origin in all vertebrates.

The mammalian ENT is a pallidal nucleus that seems to modulate motor behaviors through its projections to the lateral habenula (Shabel et al., 2012). This circuit appears to be highly conserved across the phylum, as an equivalent pallidal system has been identified in lamprey (Yáñez and Anadón, 1994; Yáñez et al., 1999; Stephenson-Jones et al., 2011, 2012, 2013). Although the ENT of rat contains both GABA and glutamate, the projection to the habenula is excitatory (Shabel et al., 2012, 2014), and this is also found in lamprey (Stephenson-Jones et al., 2013). In zebrafish, the projection from the ENT to the habenula is probably excitatory, as most ENT cells express glut2a+ in the adult (Amo et al., 2014) and larva (present results) and only a few GABAergic cells are present (Mueller and Guo, 2009). Lee et al. (2010) reported that photobleaching afferents to the habenula from the ventrolateral telencephalon caused disruption in avoidance behavior to an inescapable stressor. Although the identity of the ventrolateral nucleus was not confirmed in that study, it is likely to be the larval ENT. Consequently, the ENT is a conserved part of the basal ganglia, most probably playing a crucial role in selecting and modulating motor behaviors in response to fear and reward across vertebrates (Aizawa et al., 2011; Amo et al., 2014).

### Comparative Analysis of Other Afferents to the Habenula in Zebrafish

Apart from the ENT-habenula connection, our experiments reveal four other main neuronal populations that project to the habenula in the adult and larval zebrafish. Two of these populations (mitral cells and parapineal neurons) project asymmetrically to the habenula (dorsal right and dorsal left habenula, respectively), whereas the other two populations (rostrolateral nucleus and posterior tuberculum/posterior hypothalamic lobe) seem to project bilaterally to both habenulae, although the existence of asymmetries cannot be ruled out. In addition, the subpallium and preoptic area could send minor projections to the habenula, as shown previously in trout (Yáñez and Anadón, 1996; Folgueira et al., 2004) and goldfish (Villani et al., 1996). Finally, we also observe a minor afferent projection from the median raphe, as described previously (Lillesaar et al., 2009).

Although we offer a more detailed view of habenula connectivity in zebrafish than previously described (Aizawa et al., 2005, 2007; Hendricks and Jesuthasan, 2007; Miyasaka et al., 2009, 2014; Lee et al., 2010; Amo et al., 2014), our results are unlikely to be complete due to limitations in DiI tracing. Several lines of evidence suggest there may be additional afferents or more to investigate within the detail of the innervation by the afferent regions we have described. For instance, there are robust asymmetries in the functional response properties of left and right-sided dorsal habenular neurons (Dreosti et al., 2014; Jetti et al., 2014) that cannot be fully explained by the known asymmetric mitral cell and parapineal innervation. Furthermore, we might have expected input from the locus coeruleus and pallium. We discuss these “missing” projections in more detail below.

#### Missing Locus Coeruleus Input?

Noradrenergic fibers originating from the locus coeruleus are present in the zebrafish habenula, as revealed by dopamine-β-hydroxylase (DβH) immunohistochemistry (Ma, 1994). However, no habenulopetal neurons have been reported in the locus coeruleus in tracing studies (Hendricks and Jesuthasan, 2007; present results). Locus coeruleus neurons have profusely branched axons projecting widely in the brain, but comparatively sparse fibers reaching the habenula (Ma, 1994) and potentially, DiI tracing is not sufficiently sensitive or effective to label the sparse, fine terminal processes of the locus coeruleus neurons.

#### Missing Afferent Input in Response to Visual and Odor Stimuli?

The dorsal right habenula receives afferents from a subset of mitral cells in the olfactory bulb (Miyasaka et al., 2009, 2014; *present results*) and neuronal activity in response to odor cues is greater in right compared to left habenula (Dreosti et al., 2014; Jetti et al., 2014; Krishnan et al., 2014). The identity of odor stimuli producing responses in the asymmetrically projecting mitral cells is not clear; however, multiple studies of odor-evoked activity in the olfactory bulb and dorsal habenulae have demonstrated responses using various diverse odorants (Dreosti et al., 2014; Jetti et al., 2014; Krishnan et al., 2014). Thus it seems likely the response properties of habenular neurons to odors are highly complex (Dreosti et al., 2014; Jetti et al., 2014) and it is far from clear that all asymmetric responses could be mediated by the currently known asymmetric mitral cell input. Indeed, as neurons in both habenulae respond to odors (Dreosti et al., 2014; Jetti et al., 2014; Okamoto, 2014) there must be routes, other than via direct mitral cell innervation, for such sensory input to reach the habenulae. One possible indirect route could be via the subpallial region from which we see habenula afferents (ventral area of the subpallium (Vv); present results). This area is known to receive innervation from the olfactory bulb (Gayoso et al., 2012), although probably not from all glomeruli (Miyasaka et al., 2014).

Our results do not provide insights into the habenular afferent input that underlies the preferential activation of left-sided habenular neurons to a light flash (Dreosti et al., 2014) and other visual stimuli. Although parapineal innervation is one route by which light information reaches left habenular neurons, asymmetric habenular responses to light are present in parapineal ablated embryos and are bilateral in symmetric double-left habenulae in which the parapineal is only present on the left (Dreosti et al., 2014). These results imply that there is a probably an indirect visual pathway from the eyes to the left habenula that still remains to be identified.

#### Missing Pallial Afferents?

Hendricks and Jesuthasan (2007) described an asymmetric projection from the pallium to the dorsal right habenula. Other than afferents from mitral cells that have their origin in the pallium, we did not observe any other pallial afferents to the habenula in adult or larval zebrafish. Given the approaches we have used, we cannot unequivocally conclude that regions from which we have not observed afferent input do not project to the habenulae. Consequently, there could still be pallial afferents to the habenula, but our results are in agreement with results in other teleosts (Villani et al., 1996; Yáñez and Anadón, 1996; Yáñez et al., 1999) in which no pallial projections to the habenula have been observed. The pallial neurons identified by Hendricks and Jesuthasan (2007) are likely to be mitral cells of the olfactory bulb, we suggest this for two reasons. First, the projection described by Hendricks and Jesuthasan (2007) decussates before innervating the right habenula, as do mitral cell projections (Miyasaka et al., 2009, 2014). Second, prior to full eversion of the pallium, mitral cells are dorsally located in the developing telencephalon (Folgueira et al., 2012) in a position easily mistaken for Dm/Dp. [Authors agree with the Hendricks and Jesuthasan erratum (2011) reinterpreting pallial afferents to the habenula as mitral cells].

#### Supallium, Nucleus Rostrolateralis and Posterior Tuberculum

Our tract-tracing and transgene experiments identified a small number of cells projecting from Vv to habenula; given caveats above, we cannot be sure how complete the labeling was and hence whether the small number of labeled cells is an accurate reflection of the size of this projection. This connection may represent an ancestral part of the limbic system that seems to be conserved in teleosts, as it has been also described in trout and goldfish (Villani et al., 1996; Yáñez and Anadón, 1996). Homologies between areas of the teleost subpallium and other vertebrates (e.g., tetrapods) are uncertain because of the everted nature of the telencephalon in actinopterygii (Folgueira et al., 2012). However, Vv is proposed to be homologous to the septum in other vertebrates (Rink and Wullimann, 2004; Mueller and Wullimann, 2009) and in mammals there is a limbic projection from the septum to the medial habenula (Herkenham and Nauta, 1977; Hikosaka et al., 2008) that is probably part of anxiety and fear pathways (Yamaguchi et al., 2013). Thus, the zebrafish Vv-habenular projection could represent a limbic septo-habenular pathway, although more studies are necessary to test this hypothesis.

After DiI application to the right and left habenulae, we observe a labeled population that we tentatively identify as the thalamic rostrolateral nucleus (Saidel and Butler, 1997; Butler and Saidel, 2003; Saidel, 2013). However, as these cells are located very close to the habenula, we cannot rule out the possibility of non-specific labeling in our experiments. Despite this caveat, results in other species seem to support this connection, as potentially homologous nuclei were described in the dorsal thalamus of a shark (Giuliani et al., 2002) and a lizard (Díaz and Puelles, 1992).

A further habenulopetal population was observed in the posterior tuberculum/posterior hypothalamic lobe of zebrafish (Hendricks and Jesuthasan, 2007; present results), which is similar to that reported in trout (Yáñez and Anadón, 1996). Histaminergic neurons have been observed in this region of the zebrafish posterior tuberculum/posterior hypothalamic lobe, and conspicuous histaminergic fibers innervate the ventral region of the zebrafish habenula (Kaslin and Panula, 2001). This suggests that some of the cells retrogradely labeled from the habenula could be histaminergic. Habenulopetal cells have also been reported in the hypothalamus of lamprey (Yáñez and Anadón, 1996; Stephenson-Jones et al., 2012), lizard (Díaz and Puelles, 1992) and rat (Herkenham and Nauta, 1977), suggesting conserved hypothalamus-habenular projections in distant vertebrate groups.

## Author Contributions

MF conceived and designed the work, with inputs from KJT, TAH and SWW. KJT, TAH, JY and MF acquired and analyzed the data. All authors contributed to interpretation of data and to writing the article.

## Funding

This study was supported by Wellcome Trust (104682/Z/14/Z and 089227/Z09/Z) and EU FP-7 (ZF-HEALTH) Grants to SW (Orcid ID 0000–0002–8557–5940; loop: s.wilson@ucl.ac.uk); BBSRC funding (BB/H012516/1) to SWW and TAH (Orcid ID 0000-0003-2921-0004).

## Conflict of Interest Statement

The authors declare that the research was conducted in the absence of any commercial or financial relationships that could be construed as a potential conflict of interest.

## Abbreviations

ac: anterior commissure
CC: cerebellar corpus
D: diffuse nucleus of the hypothalamus
dENT: dorsal entopeduncular nucleus
dHbL: lateral subnucleus of the dorsal habenula
dHbM: medial subnucleus of the dorsal habenula
Dl: lateral area of the pallium
Dm: medial area of the pallium
Dp: posterior area of the pallium
ENT: entopeduncular nucleus
fr: fasciculus retroflexus
GL: glomerular layer of the olfactory bulb
Hb: habenula
Hd: dorsal zone of the periventricular hypothalamus
HL: lateral hypothalamic lobe
lfb: lateral forebrain bundle
lHb: left habenula
ML: mitral layer of the olfactory bulb
MR: median raphe
OB: olfactory bulb
och: optic chiasm
OT: optic tectum
Otr: optic tract
P: pineal
Pal: pallium
PG: preglomerular complex
PHL: posterior hypothalamic lobe
PO: preoptic region
Pp: parvocellular preoptic nucleus
PP: parapineal
PT: posterior tuberculum
PTh: prethalamus
PTN: posterior tuberal nucleus
rHb: right habenula
RL: rostrolateral nucleus
SG: secondary gustatory nucleus
StMed: stria medullaris
Tel: telencephalic lobes
Th: thalamus
tHC: tract of the habenular commissure
TL: longitudinal torus
TLa: lateral torus
TS: semicircular torus
V: subpallium
VC: valvula cerebelli
Vd: dorsal area of the subpallium
vENT: ventral entopeduncular nucleus
vHb: ventral habenula nucleus
Vl: lateral area of the subpallium
Vs: supracommissural area of the subpallium
Vv: ventral area of the subpallium
ZLI: zona limitans intrathalamica.
